# Political economy of Thailand's tax-financed universal coverage scheme

**DOI:** 10.2471/BLT.19.239343

**Published:** 2019-11-18

**Authors:** Viroj Tangcharoensathien, Jadej Thammatach-aree, Woranan Witthayapipopsakul, Shaheda Viriyathorn, Anond Kulthanmanusorn, Walaiporn Patcharanarumol

**Affiliations:** a International Health Policy Program, Ministry of Public Health, Tivanon Road, Muang District, Nonthaburi Province 11000, Thailand.; b National Health Security Office, Nonthaburi, Thailand.

## Abstract

**Problem:**

The challenge of implementing contributory health insurance among populations in the informal sector was a barrier to achieving universal health coverage (UHC) in Thailand.

**Approach:**

UHC was a political manifesto of the 2001 election campaign. A contributory system was not a feasible option to honour the political commitment. Given Thailand’s fiscal capacity and the moderate amount of additional resources required, the government legislated to use general taxation as the sole source of financing for the universal coverage scheme.

**Local setting:**

Before 2001, four public health insurance schemes covered only 70% (44.5 million) of the 63.5 million population. The health ministry received the budget and provided medical welfare services for low-income households and publicly subsidized voluntary insurance for the informal sector. The budgets for supply-side financing of these schemes were based on historical figures which were inadequate to respond to health needs. The finance ministry used its discretionary power in budget allocation decisions.

**Relevant changes:**

Tax became the sole source of financing the universal coverage scheme. Transparency, multistakeholder engagement and use of evidence informed budgetary negotiations. Adequate funding for UHC was achieved, providing access to services and financial protection for vulnerable populations. Out-of-pocket expenditure, medical impoverishment and catastrophic health spending among households decreased between 2000 and 2015.

**Lessons learnt:**

Domestic government health expenditure, strong political commitment and historical precedence of the tax-financed medical welfare scheme were key to achieving UHC in Thailand. Using evidence secures adequate resources, promotes transparency and limits discretionary decision-making in budget allocation.

## Résumé

**Problème:**

La difficulté de mettre en œuvre une assurance maladie contributive dans les populations du secteur informel faisait obstacle à la couverture sanitaire universelle (CSU) en Thaïlande.

**Approche:**

La CSU était au cœur de la campagne électorale de 2001. Pour respecter les engagements politiques, la mise en place d'un système contributif n'était pas envisageable. Compte tenu de la capacité fiscale du pays et des ressources supplémentaires nécessaires, relativement modérées, le gouvernement a voté le recours à l'imposition générale comme seule source de financement du régime de couverture universelle.

**Environnement local:**

Avant 2001, quatre régimes publics d'assurance maladie couvraient seulement 70% (44,5 millions) de la population (63,5 millions d'habitants). Le ministère de la Santé a reçu le budget et a mis en place des services de protection médicale pour les ménages à faible revenu ainsi qu’une assurance facultative subventionnée par l'État dans le secteur informel. Les budgets de financement « côté offre » de ces régimes reposaient sur des chiffres historiques et ne permettaient pas de répondre aux besoins en matière de santé. Le ministère des Finances a usé de son pouvoir discrétionnaire pour orienter les décisions concernant l'affectation du budget.

**Changements significatifs:**

L'impôt est devenu la seule source de financement du régime de couverture universelle. Les négociations budgétaires ont gagné en transparence, en faisant intervenir différentes parties prenantes et en s'appuyant sur des éléments probants. Un financement adéquat a pu être dégagé pour la CSU, ce qui a permis aux populations vulnérables d'accéder à des services et de bénéficier d'une protection financière. Les dépenses directes, l'appauvrissement pour raison médicale et les dépenses de santé exorbitantes des ménages ont diminué entre 2000 et 2015.

**Leçons tirées:**

Les dépenses du gouvernement pour la santé, un engagement politique fort ainsi que les précédents historiques du régime de protection médicale financé par l'impôt ont été essentiels pour parvenir à la CSU en Thaïlande. Le recours à des éléments probants permet d'obtenir des ressources adéquates, favorise la transparence et limite la prise de décisions discrétionnaires concernant l'affectation du budget.

## Resumen

**Situación:**

El reto de implementar un seguro de salud contributivo entre las poblaciones del sector informal era un obstáculo para lograr la cobertura sanitaria universal (universal health coverage, UHC) en Tailandia.

**Enfoque:**

La UHC fue un manifiesto político de la campaña electoral de 2001. Un sistema contributivo no era una opción viable para cumplir el compromiso político. Dada la capacidad fiscal de Tailandia y la cantidad moderada de recursos adicionales necesarios, el Gobierno legisló para utilizar los impuestos generales como única fuente de financiación del plan de cobertura universal.

**Marco regional:**

Antes de 2001, cuatro planes de seguro de salud públicos cubrían solo el 70 % (44,5 millones) de los 63,5 millones de habitantes. El Ministerio de Salud recibe el presupuesto y presta servicios de asistencia médica a los hogares de bajos ingresos y subsidia públicamente los seguros voluntarios para el sector informal. Los presupuestos para la financiación de la oferta de estos planes se basaron en cifras históricas que eran insuficientes para responder a las necesidades sanitarias. El Ministerio de Hacienda utilizó su poder discrecional en las decisiones de asignación presupuestaria.

**Cambios importantes:**

El impuesto se convirtió en la única fuente de financiación del plan de cobertura universal. La transparencia, la participación de múltiples partes interesadas y el uso de pruebas informaron las negociaciones presupuestarias. Se logró una financiación adecuada para la UHC, proporcionando acceso a los servicios y protección financiera para las poblaciones vulnerables. Los gastos de bolsillo, el empobrecimiento de los servicios médicos y el gasto catastrófico en salud de los hogares disminuyeron entre 2000 y 2015.

**Lecciones aprendidas:**

El gasto sanitario del gobierno nacional, el fuerte compromiso político y la precedencia histórica del plan de bienestar médico financiado por los impuestos fueron fundamentales para lograr la UHC en Tailandia. El uso de pruebas asegura recursos adecuados, promueve la transparencia y limita la toma de decisiones discrecionales en la asignación presupuestaria.

## ملخص

**المشكلة:**

كان تحدي تنفيذ التأمين الصحي القائم على المساهمة بين السكان في القطاع غير الرسمي، يمثل عائقًا أمام تحقيق التغطية الصحية الشاملة (UHC) في تايلند.

**الأسلوب:**

كانت التغطية الصحية الشاملة عبارة عن بيان سياسي للحملة الانتخابية لعام 2001. لم يكن نظام المساهمة خياراً ممكناً لاحترام الالتزام السياسي. في ظل القدرة المالية لتايلند والحجم المعتدل من الموارد الإضافية المطلوبة، وضعت الحكومة تشريعات لاستخدام الضرائب العامة كمصدر وحيد لتمويل خطة التغطية الشاملة.

**المواقع المحلية:**

قبل عام 2001، كانت هناك أربع خطط للتأمين الصحي العام غطت 70% فقط (44.5 مليون) من 63.5 مليون نسمة. حصلت وزارة الصحة على الميزانية وقدمت خدمات الرعاية الطبية للأسر ذات الدخل المنخفض، وقدمت كذلك دعماً شعبياً للتأمين التطوعي للقطاع غير الرسمي. اعتمدت ميزانيات تمويل الإمداد لهذه الخطط على أرقام تاريخية لم تكن كافية لتلبية الاحتياجات الصحية. لجأت وزارة المالية لسلطتها التقديرية في قرارات تخصيص الميزانية.

**التغيّرات ذات الصلة:**

أصبحت الضريبة المصدر الوحيد لتمويل خطة التغطية الشاملة. وأدت الشفافية وإشراك أصحاب المصلحة المتعددين واستخدام الأدلة، إلى توجيه مفاوضات الميزانية. تم تحقيق التمويل الكافي للتغطية الصحية الشاملة، مع إتاحة إمكانية الوصول إلى الخدمات والحماية المالية للسكان من الفئات المعرضة للخطر. انخفض الإنفاق غير المباشر، والإفقار الطبي والإنفاق الصحي في حالات الكوارث بين الأسر، بين عامي 2000 و2015.

**الدروس المستفادة:**

إن كل من الإنفاق الحكومي المحلي على الصحة، والالتزام السياسي القوي، والأسبقية التاريخية لخطة الرعاية الطبية الممولة من الضرائب، كانت عوامل أساسية نحو تحقيق التغطية الصحية الشاملة في تايلند. إن استخدام الأدلة يضمن تأمين الموارد الكافية، ويعزز الشفافية، ويحد من اتخاذ القرارات التقديرية في تخصيص الميزانية.

## 摘要

问题 如何在非正式部门人口中实施自缴型健康保险是泰国实现全民健康保险 (UHC) 的一大障碍。

方法 实现全民健康保险 (UHC) 是 2001 年竞选活动的竞选宣言。自缴制度不是履行竞选宣言的可行方法。鉴于泰国的财政能力和所需有限的其他资源,泰国政府立法规定将普通税收作为全民健康计划的唯一资金来源。

当地状况2001 年以前,在 6350 万人口中,四大公共健康保险计划只覆盖了 70%(4450 万)的人口。卫生部收到预算,为低收入家庭提供医疗福利服务,并为非正式部门提供公费补贴的自愿保险。这些计划的供应方融资预算是根据历史数据编制的,而且这些数据不足以满足健康需求。财政部在预算分配决策中使用了它的自由裁量权。

相关变化 税收成为为全民健康计划的唯一资金来源。透明度、多方利益相关方的参与和证据的使用为预算谈判提供了参考信息。为全民健康覆盖提供了充足的资金,为弱势群体提供了服务和财政保护的机会。2000 年至 2015 年期间,自付支出、医疗贫困和家庭灾难性卫生支出有所减少。

经验教训 泰国政府的健康支出、强有力的政治承诺和依靠税收的医疗福利计划的长久惯例是实现全民健康保险的关键。使用证据可以确保充足的资源,提高透明度,并限制预算分配中的自由裁量权。

## Резюме

**Проблема:**

Сложности внедрения основанной на взносах программы медицинского страхования среди групп населения в неформальном секторе препятствовали достижению всеобщего охвата услугами здравоохранения в Таиланде.

**Подход:**

Всеобщий охват услугами здравоохранения входил в политический манифест избирательной кампании 2001 года. Система взносов не была приемлемым вариантом с точки зрения выполнения политических обязательств. Учитывая финансовые возможности Таиланда и небольшой объем необходимых дополнительных ресурсов, правительство приняло закон об использовании общей системы налогообложения в качестве единственного источника финансирования системы всеобщего охвата услугами здравоохранения.

**Местные условия:**

До 2001 года четыре существующие программы государственного медицинского страхования охватывали лишь 70% (44,5 млн) из 63,5 млн населения. Министерство здравоохранения получило бюджет и обеспечило медицинское обслуживание и социальную помощь семьям с низким уровнем дохода и субсидируемую государством программу добровольного страхования для неформального сектора. Объем бюджетов для финансирования данных программ основывался на данных за прошлые периоды, финансирование в которые было недостаточным для удовлетворения потребностей здравоохранения. Министерство финансов воспользовалось своими широкими полномочиями для принятия решений о распределении бюджетных средств.

**Осуществленные перемены:**

Единственным источником финансирования системы всеобщего охвата услугами здравоохранения стали налоги. Обсуждения бюджета основывались на информационной открытости, участии многих заинтересованных сторон и использовании фактических данных. Было обеспечено адекватное финансирование системы всеобщего охвата услугами здравоохранения, обеспечивающее доступ к услугам и финансовую защиту наиболее уязвимым группам населения. Расход наличных средств, обнищание из-за расходов на медобслуживание и катастрофические расходы на медицинское обслуживание в семьях сократились в период с 2000 по 2015 год.

**Выводы:**

Внутренние государственные расходы на здравоохранение, твердая политическая позиция и наличие исторического прецедента существования финансируемой из налоговых поступлений системы медицинского обслуживания и социальной помощи стали ключевыми факторами для достижения всеобщего охвата услугами здравоохранения в Таиланде. Использование фактической информации обеспечивает выделение адекватных ресурсов, способствует прозрачности и ограничивает принятие дискреционных решений при распределении бюджета.

## Introduction

Though there are several approaches to financing universal health coverage (UHC), tax-based schemes have been advocated by the World Health Organization and international development partners. Taxation can be a progressive method of raising government funds for health (when richer people pay more than poorer people) and has lower administrative costs and is more feasible than contributory health insurance schemes.^1^ The challenge of enforcing mandatory insurance premiums for health care among populations in the informal sector is a major barrier to achieving UHC.^2^

Increasing domestic tax revenue is especially important for achieving UHC in countries with low tax bases. For each 100 United States dollars (US$) per capita annual increase in tax revenue results in a US$ 9.86 increase (95% confidence interval, CI: 3.92–15.8) in government spending on health.^3^ Here we report how historical precedence and the political situation in Thailand paved the way for taxation as the sole source of financing for the universal coverage scheme. 

## Local setting

Thailand’s medical welfare scheme for low-income households was launched in 1975 and later extended to cover elderly people, children younger than 12 years and disabled people. The voluntary health card scheme, launched in 1984 for non-poor households in the informal sector, was financed by premium contributions. Aiming to increase coverage, in 1994 the government began subsidizing 50% of the premiums.^4^ In 1980, the government legislated regulation on the civil servant medical benefit scheme as a non-contributory tax-funded scheme to cover government officers, pensioners and their dependents. Social health insurance, legislated in 1990 for private sector employees, was financed through payroll tax with equal contributions from employers, employees and the government. Financing social health insurance was categorized as public financing.^4^

**Fig. 1:**
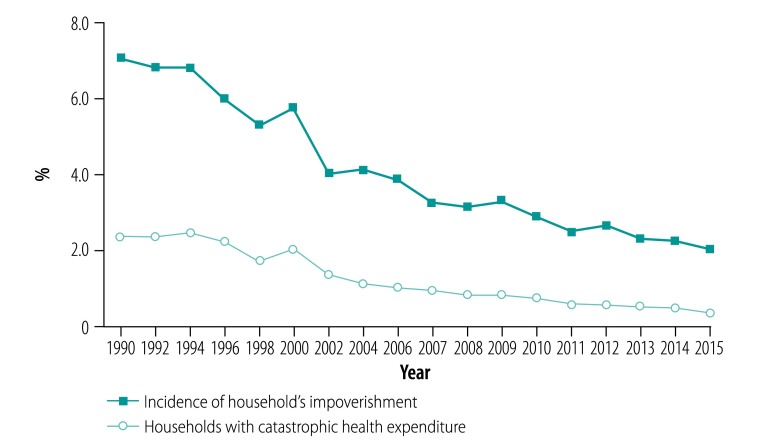
Incidence of catastrophic health expenditure and household impoverishment, Thailand, 1990–2015

Source: Authors’ analysis using socioeconomic surveys conducted by Thailand’s National Statistical Office, 1990–2015. Data used for the calculation of catastrophic health expenditure and household impoverishment are available in the authors’ data repository.^6^

There were three budgetary challenges to these schemes. First, budget allocation to the medical welfare scheme and voluntary health insurance was based on historical figures with minimal annual increases. The per capita budget support to the medical welfare scheme increased from 225 Thai baht (THB) in 1995^5^ to 273 THB in 2001 (the conversion rate was US$ 1 at 30.3 THB in 2019). Subsidies to voluntary health insurance were 500 THB per household of four members, equivalent to 125 THB per capita. The budget was inadequate and did not reflect the total cost of health care provision, leaving the shortfalls to be borne by out-of-pocket payments from the members. In 2000, out-of-pocket payments were 34.2% (US$ 21.2) of the current health expenditure of US$ 62 per capita. The incidence of medical impoverishment was 2.0% (0.32 million out of 16.1 million households) and of catastrophic health spending (> 10% of total household expenditure) was 5.7% (0.92 million households; Fig. 1).

Second, the finance ministry was responsible for allocating health service budgets to health ministry-owned facilities at sub-district, district and provincial levels. The allocation applied incremental increases, and was often decided by the discretionary powers of the finance ministry, particularly on the capital budget. The finance ministry was responsible for reviewing all competing budget proposals against government priorities and submitting budget ceilings for the prime minister’s approval. The finance ministry was therefore able to influence final decisions on the ministerial budget ceiling.^7^

Third, these multiple budget flows to health facilities confused accountability among the health facilities (the recipients of funds), the four health insurance funds, the health ministry and the citizens (the taxpayers).

## Approaches

UHC was a part of the political manifesto of the *Thai Rak Thai* party, who were able to form a coalition government in 2001.^8^ Prior to the election, discussions within the party were in favour of collecting 100 THB monthly premiums from the uninsured. However, for several reasons, the proposal was withdrawn a few weeks before the election.^9^

First, a financial analysis showed that combining all existing budget streams (health ministry annual budget, medical welfare scheme and voluntary health insurance scheme) would require a moderate additional budget to implement a non-contributory scheme and this amount was within the prime minister’s power to mobilize.^11^ Second, the voluntary health insurance scheme had adverse selection of members, because healthy people did not join and the high proportion of sick members undermined the financial viability of the scheme.^10^ Third, collecting and enforcing premium payments among those working in the informal sector with erratic and seasonal incomes was politically and technically difficult. Failure of people to contribute would interrupt their membership and hinder access to care.

Opposition parties in parliament raised concerns that tax revenues for health should not benefit richer people who were able to pay their own medical bills. Civil society organizations rejected this argument by highlighting that an entitlement to public health services, even for the rich, was enshrined by Article 52 of the 1997 Thai Constitution.^11^ However, some university academics preferred premium contributions, arguing that a tax-financed scheme might be jeopardized by changing government policies and funding interruptions. These criticisms did not change the government’s decision to use taxation, an outcome which could be explained by stakeholder theory.^12^ In this case, the *Thai Rak Thai* party qualified as the dominant stakeholder. The party had constitutional legitimacy because UHC endorses the right to health for everyone. The party also had both legislative power through a majority in parliament and executive power to mobilize additional budgets. The policy on UHC was socially acceptable and a matter of urgency as a political promise to implement within a year. With the combination of power, legitimacy and urgency, the party became the definitive stakeholder and was in position to win over opponent stakeholders. Ultimately, the universal coverage scheme was designed to be financed wholly by general taxation and this was legislated into Article 39(1) of the 2002 National Health Security Act.^13^

An advantage was that health-care providers, one of the key stakeholders, did not oppose the reform. The overall budget for the universal coverage scheme increased substantially from the 273 and 125 THB per capita (total population for medical welfare: 18.4 million) and voluntary health insurance scheme (total population: 14.9 million) in 2001 to 1202 THB per capita universal coverage scheme member in 2002.

In 2001, the budget allocation to the health ministry for publicly-financed health services was 26.5 billion THB. The total resources required for a universal coverage scheme was estimated based on 1202 THB^14^ per capita multiplied by 47 million universal coverage scheme members, equivalent to 56.5 billion THB. The additional budget, 30 billion THB (a funding gap of between 56.5 and 26.5 billion THB), was within the capacity of the prime minister to mobilize. To prevent double funding to public health facilities, the supply-side budget was terminated and included in annual budgets of the universal coverage scheme.

The government has adopted the principle of per capita budgeting for the scheme. The annual per capita budget was the product of the related unit cost of services and quantity of services provided as measured by utilization rates. The total budget requested to the government, through the finance ministry, was the product of per capita budget and the total number of universal coverage scheme members. Budgetary process, based on objective evidence, was managed by a multistakeholder subcommittee, which prevented the use of discretionary power by the finance ministry.

## Relevant changes

All Thai citizens were entitled to one of three non-competing public schemes. The newly implemented universal coverage scheme covered the population who were not beneficiaries of the existing schemes. Individuals’ enrolment into health insurance schemes were automatically switched based on the changes in their eligibility status, such as age and employment. For example, children of civil servant medical benefit scheme members would be entitled to the universal coverage scheme as soon as they turned 20 years old; universal coverage scheme members who became employed by the private sector would be automatically enrolled into payroll-tax financed social health insurance.

The universal coverage scheme offered a comprehensive benefit package inclusive of outpatient, inpatient and emergency care, high-cost care, dental services, health promotion and disease prevention, and all medicines in the national list of essential medicines. Closed-end provider payments were adopted, notably capitation and diagnostic-related groups, and these methods improved efficiency and contained costs. A primary-care gate-keeping system was also adopted.^4^ Copayments of US$ 1 per visit or per admission (later copayment was ended in 2008) boosted financial protection. Out-of-pocket payments of health expenditure decreased to 11.3% (US$ 25.2) of the current per capita health expenditure (US$ 221.9) in 2016.^15^ By 2015, the incidence of household’s medical impoverishment had fallen to 0.3% (71 524 of 21.3 million households) when the national poverty line was applied and the incidence of catastrophic health spending also decreased to 2.0% (427 808 of 21.3 million households; Fig. 1).

In addition, the universal coverage scheme had reduced the infant mortality gaps between poorer and richer provinces between 2000 and 2002 because of increased access to health care among the poor.^16^

Thailand’s UHC index in 2015 was 75 (on a scale of 0–100) with a low level of unmet health care needs, a steady decline in all-cause mortality between 2001–2014, and reduced inequality of adult mortality across geographical areas.^4^^,^^17^ Engagement by multistakeholders in the subcommittee promotes transparency of the budgetary process.^18^ Table 1 compares relevant changes before and after the introduction of the scheme.

**Table 1:** Relevant changes in health care before and after introduction of the universal coverage scheme in Thailand in 2002

Relevant changes	Before universal coverage scheme in 2001	After universal coverage scheme in 2016
**Population coverage**	Only 70% (44.5 out of 63.5 million) of the Thai population were covered by many fragmented health schemes	More than 99% (68.2 out of 68.9 million) of the Thai population were covered by the three main public health schemes. The new scheme covers the majority, about 51.7 million (75%) of the population
**Budgetary process**	Parallel funding, with annual supply-side budget allocation and funding for the medical welfare and voluntary insurance schemes	Termination of supply-side budget allocation
	Historical incremental budget increases	Full cost subsidies to a comprehensive package
	Full cost of services for the two schemes was not reflected in government budgets	Evidence-based budget estimates are based on service utilization rates and unit costs
	Budget allocation was at the discretion of the finance ministry	Multistakeholder financing subcommittee ensures transparency and limited room for discretion by the finance ministry
**Governance and relationships between providers and funding agencies **	In a public integrated model whereby the health ministry played both financing- and service-provision roles for the medical welfare and voluntary insurance schemes	Splitting the role of purchaser and provider, the health ministry maintains a service-provision role, National Health Security Office, which manages the new scheme, is responsible for strategic purchasing function
**Financial protection**		
Current health expenditure, THB millions	161 752.41	547 735.15
General government expenditure, THB millions	801 690.44	2 737 009.17
Out-of-pocket expenditure, THB millions (% of current health expenditure)	54 977.39 (33.9)	62 144.05 (11.3)
Domestic general government health expenditure, THB millions (% of general government expenditure)	88 987.64 (11.1)	416 025.39 (15.2)
Domestic general government health expenditure, THB millions (% of current health expenditure)	88 987.64 (55.0)	416 025.39 (76.0)

THB: Thai baht.

Note: the conversion rate in 2019 was 1 United States dollars at 30.3 THB

Source: Financial protection data are from the National Health Account of Thailand, 2019.^19^

## Lesson learnt

Domestic government health expenditure was key towards achieving UHC in Thailand due to the large population working in the informal sector. Political commitment and historical precedence of tax-financed medical welfare scheme were also important. However, a comprehensive benefit package with nominal copayments can significantly reduce out-of-pocket expenditure and improve financial protection. The participatory use of evidence in budgetary processes limits discretionary decisions by ministries and promotes transparency and accountability. Finally, sustained political commitment and civil society engagement are key contributing factors (Box 1). The Thai universal coverage scheme has survived two decades through rival governments and a climate of political conflict. A network of bureaucrats who mobilized resources in the bureaucracy, political parties, civil society and international organizations helped institutionalize the universal coverage scheme in the face of broader professional dissent and political conflicts.^17^

Box 1 **Summary of main lessons learnt**

• Domestic government health expenditure, political commitment and a tax-financed scheme, which promotes greater equity, were essential to achieving universal health coverage in Thailand.

• A shift from supply-side to demand-side budgeting and the use of evidence secures adequate resources, promotes transparency, limits discretionary budget allocation and improves accountability to citizens.

• A comprehensive benefit package can reduce out-of-pocket expenditure and improve financial protection for the population.
